# Quantitative stakeholder-driven assessment of radiation protection issues via a PIANOFORTE online survey

**DOI:** 10.1007/s00411-024-01084-1

**Published:** 2024-07-17

**Authors:** Veronika Groma, Balázs Madas, Florian Rauser, Mandy Birschwilks, Andreas Blume, Almudena Real, Rein Murakas, Boguslaw Michalik, Isabel Paiva, Tone-Mette Sjømoen, Alan H. Tkaczyk, Jelena Mrdakovic Popic

**Affiliations:** 1 Environmental Physics Department, HUN-REN Centre for Energy Research, Budapest, Hungary; 2 Federal Office for Radiation Protection, BfS, Germany; 3 Research Centre on Energy, Environment and Technology, CIEMAT, Madrid, Spain; 4 Faculty of Social Sciences, University of Tartu, Tartu, Estonia; 5 Faculty of Arts and Humanities, University of Tartu, Tartu, Estonia; 6 Rein Murakas Consulting, Tartu, Estonia; 7 Silesian Centre for Environmental Radioactivity, Central Mining Institute, Katowice, Poland; 8 Center for Nuclear Sciences and Technologies, Department of Nuclear Engineering and Sciences, Instituto Superior Técnico, University of Lisbon, Lisbon, Portugal; 9 Norwegian Radiation and Nuclear Safety Authority, Oslo, Norway; 10 Institute of Technology, University of Tartu, Tartu, Estonia

**Keywords:** Radiation protection, Risk perception, E-survey, Stakeholder engagement, Positive Matrix factorization (PMF)

## Abstract

To enhance stakeholder engagement and foster the inclusion of interests of citizens in radiation protection research, a comprehensive online survey was developed within the framework of the European Partnership PIANOFORTE. This survey was performed in 2022 and presented an opportunity for a wide range of stakeholders to voice their opinions on research priorities in radiation protection for the foreseeable future. Simultaneously, it delved into pertinent issues surrounding general radiation protection. The PIANOFORTE e-survey was conducted in the English language, accommodating a diverse range of participants. Overall, 440 respondents provided their insights and feedback, representing a broad geographical reach encompassing 29 European countries, as well as Canada, China, Colombia, India, and the United States. To assess the outcomes, the Positive Matrix Factorization numerical model was applied, in addition to qualitative and quantitative assessment of individual responses, enabling the discernment of four distinct stakeholder groups with varying attitudes. While the questionnaire may not fully represent all stakeholders due to the limited respondent pool, it is noteworthy that approximately 70% of the participants were newcomers to comparable surveys, demonstrating a proactive attitude, a strong willingness to collaborate and the necessity to continuously engage with stakeholder groups. Among the individual respondents, distinct opinions emerged particularly regarding health effects of radiation exposure, medical use of radiation, radiation protection of workers and the public, as well as emergency and recovery preparedness and response. In cluster analysis, none of the identified groups had clear preferences concerning the prioritization of future radiation protection research topics.

**Supplementary Information:**

The online version contains supplementary material available at 10.1007/s00411-024-01084-1.

## Introduction

The PIANOFORTE partnership, formally known as the ‘Partnership for European research in radiation protection and detection of ionising radiation: towards a safer use and improved radiation protection of the environment and human health’, is a project funded by the European Commission’s Euratom Programme. Commencing its activities in June 2022, this project unites a large consortium of far more than fifty European organizations originating from 22 EU member states, Norway and the UK.

The key objective of the PIANOFORTE partnership is to provide support and facilitate the consolidation of research and development within the domain of radiation protection (RP). By doing so, it empowers national authorities to foster advancements in regulatory practices by employing new knowledge, innovative methodologies and technologies, and enhancing skills in this field. Equally important, the partnership aims to bridge existing knowledge gaps, address societal concerns, and face new problems related to radiation protection.

One of the partnership’s pillars is Stakeholder Engagement, and a work package (WP3) is dedicated to this activity. A primary focus of the stakeholder engagement initiative is to create meaningful connections among diverse stakeholders’ networks, both within and outside the radiation protection community to ensure that the outcomes of this research directly impact and improve the lives of all European citizens.

To actively engage stakeholders, one of the key activities is to collect their opinions about the radiation protection system, existing criticisms, considerations and expectations on possible improvements and to track this stakeholder engagement in time. To collect such information, PIANOFORTE efforts focused first on the rapid development and implementation of a dedicated electronic survey. Such an approach has proven to be informative in a previous public survey conducted under the auspices of the European Joint Programme (EJP) CONCERT (Monaca et al. 2021) as well as in the European H2020 RadoNorm project (Perko et al., 2022).

The results from the CONCERT survey in 2017 indicated that participants (being higher educated − 77%- and with a background in natural sciences − 85%) were reasonably satisfied with the existing information pertaining to ionising radiation risks (Monaca et al. 2021). However, results also highlighted the pressing need for three key improvements. Firstly, increasing the understanding of radiation protection concepts is crucial, particularly among non-professionals. A notable example can be drawn from the CONCERT 2017 survey, where respondents were questioned about their knowledge of radiation protection. Questions such as whether the human body emits radiation or if plants grown near nuclear power plants are safe for consumption revealed that nearly 45% of respondents provided incorrect answers or responded with ‘I don’t know/no answer.’ This underlines the importance of promoting a deeper understanding among a greater variety of stakeholders, even among those working in the domain of radiation protection. Secondly, the survey results emphasized the necessity to further explore the information availability and development of knowledge, again across different stakeholder groups, over time. It is crucial to continually assess and update the information provided to stakeholders to ensure its accuracy and relevance. Lastly, the survey findings underscored the significance of improving communication channels and stakeholder engagement in knowledge creation and dissemination. This entails enhancing the collaboration and active involvement of stakeholders in various stages of research projects, including those funded by the European Union.

Recent studies have revealed that for certain radiation protection topics weak communication with relevant stakeholders (such as industry operators, managers etc.) led to deeper reluctance to engage with the topic (Mrdakovic Popic et al. 2023). Turcanu et al. 2020 has revealed that prescriptions and approaches to stakeholder engagement can be enhanced by recognizing the normative and substantive justifications for engagement. Furthermore, acknowledging the significance of informal and citizen-led engagement is vital. Additionally, it is advisable to incorporate more systematic methods for stakeholder engagement in the development and assessment of national policies. In cases where opinions diverge the idea of this survey is not to “average” these opinions out, but to outline that radiation protection and specifically radiation protection research must always remain inclusive and bring together insights from several disciplines to allow a dialogue in a common language.

Radiation protection stakeholders do not share identical interests or experience the same impacts based on updated scientific evidence or new and updated legislations. Identifying the perspectives of various stakeholder groups can ensure that a plurality of voices is being considered. Further, it simplifies the management of communication challenges and helps to inquire for contributions from stakeholders.

At the start, the PIANOFORTE partnership developed an online questionnaire to map stakeholders and to prepare these networks for direct engagement through getting their opinion on different radiation protection topics. In this survey the main objective was to identify where networking and communication can be enhanced and to evaluate stakeholders’ linkage with, and involvement in, significant international initiatives relevant to radiation protection research. This paper offers a comprehensive overview of the initial PIANOFORTE partnership public survey, presenting insights into its structure, implementation, dissemination, results, analysis, and key conclusions.

## Materials and methods

The survey was designed to explore issues within the field of general radiation protection that require further research and to assess opportunities for enhancing stakeholders’ knowledge about ionising radiation. It targeted a wide range of stakeholder, which are listed in detail in Table 1.The survey was created and launched in a digital format that allowed anyone to easily enter their opinions within a reasonable timeframe of approximately 15–30 min. This condensed format was chosen to enhance stakeholder participation rates and enable potential follow-ups on a regular time scale in the future.

### Survey design

This survey was not conducted or designed to be representative of a specific part of the population (or that population at a whole). Instead, the “ensemble of opportunity that arose” reflects stakeholders with strong interest in the activities of PIANOFORTE and reflects an outcome more than a design choice of the survey.

The survey was designed as a follow-up to the previous European RP partnership EJP CONCERT survey, with results presented in Monaca et al. 2021. Insights from the 2020 survey informed the structure of the current survey, aligning with the main aims and objectives of the PIANOFORTE partnership. Social scientists reviewed the survey design, from consent to specific questions on stakeholders’ opinions and concerns about RP topics.

To avoid bias, the authors considered potential sampling and response biases. After defining the survey goals based on the previous EJP CONCERT survey and PIANOFORTE objectives, the survey was distributed to various stakeholder groups interested in RP issues. Equal opportunities were provided to different stakeholder groups, and they were encouraged to participate through meetings and emails. This effort was coordinated across all European countries via PIANOFORTE contact points and the main partnership coordinator.

The survey was shorter than previous ones in Projects STAR and CONCERT, with clear questions to engage diverse participant groups and elicit accurate, thoughtful responses. Questions were grouped by relevance to maintain participant focus.

The survey encompassed three categories of questions. First, participants were requested to consent to filling in the survey, provide information regarding their stakeholder group affiliation, name, profession, email, and country (for geographical distribution analysis). While the initial consent question was mandatory, the remaining questions in this section were made non-mandatory to satisfy data protection and ethical considerations. Secondly, PIANOFORTE partnership-related questions were asked focusing on its main features and activities. Respondents were asked in this part about the identified research priorities relating to research and innovation project funding, expected stakeholder engagement within the partnership, and preferred methods of receiving updates on the partnership’s outcomes. Thirdly, radiation protection questions were addressed in dedicated section, covering essential radiation protection topics and issues, which were of potential interest or concern to a diverse range of stakeholders.

The questions of the survey are provided in the supplementary material.

### Survey tool

The survey was conducted on the SurveyMonkey platform (https://www.surveymonkey.com/), which is a cloud-based survey platform that empowers users to easily generate, distribute, and analyse surveys. With SurveyMonkey, users have the flexibility to email survey links directly to respondents or share them on their websites and social media platforms, effectively boosting response rates. A detailed analysis if the choice of this tool reflects an implicit language and region bias was not performed, due to the nature that this language and regional bias is probably much smaller than the one introduced by the choice to only ask in English.

### Dissemination of the survey

The survey was launched in November 2022, with a response period of two months. To ensure wide participation, the survey link was distributed via email to an extensive list of contacts, including individuals from national and international organizations, researchers, regulators, implementer groups, and members of the public. A total of 990 contacts across European countries, including both participants and non-participants of the PIANOFORTE partnership, were invited to fill in the survey. These contacts were encouraged to further share the survey link with other relevant individuals. In addition to personal emails, the survey was also promoted on the PIANOFORTE webpage and on various social media platforms. The dissemination strategy placed a particular emphasis on engaging PIANOFORTE Beneficiary and Associated Partners organizations from countries involved in PIANOFORTE, as well as members of the six European radiation protection research platforms (ALLIANCE, EURADOS, EURAMED, MELODI, NERIS, SHARE). Their active involvement and support played a crucial role in reaching a broad and diverse audience in the different countries. However, it is of high significance to highlight that our primary target stakeholder groups do not include the general public or communities living near areas impacted by ionizing radiation, such as nuclear power plants.

### Limitations of the survey

The study has a few potential limitations. Firstly, there was a lack of control over the response rate. General reminders might not always be the most effective approach. Secondly, the quality of responses was affected by the opportunity of producing incomplete questionnaires and data gaps, which can significantly limit the number of usable responses. Another limitation is the lack of disclosure of respondents’ backgrounds, which could provide an even more clear understanding of their answers. While such questions have been developed for several international surveys, the length of the questionnaire and the need to guarantee anonymity limit their use. The survey responses were analysed with consideration of these potential biases.

### Data analysis

Initially, the responses to the questions were evaluated individually, analysing each answer separately. Subsequently, our objective was to identify opinion groups based on the collected data. Through the individual evaluation of the questions, we gain valuable insights into the opinions of stakeholders and their respective priorities. This allows us to generate an overview and assess the ranking of different aspects based on the stakeholders’ feedback.

Additionally, by employing a clustering approach, we can identify groups of individuals with similar attitudes and preferences and measure the prevalence and significance of these clusters within the respondent pool. By analysing the collected data, we can uncover patterns and similarities among stakeholders, enabling a deeper understanding of shared perspectives and priorities. This clustering process helps us to identify commonalities and variations in opinions, facilitating the identification of key trends and themes that emerge within the stakeholder community.

Furthermore, assessing the size of these opinion clusters provides valuable information about the prevalence and significance of certain viewpoints or preferences. It allows us to quantify the relative representation and influence of each cluster, providing insights into the diversity and distribution of opinions within the stakeholder population.

Positive Matrix Factorization (PMF) is a data evaluation technique used in various fields, including recommender systems, image analysis, and environmental science (Chu & Plemmons 2005). It has been shown that this numerical method is an effective tool in pattern recognition, data mining (Wang & Zhang 2013) and community discovery (Wang et al. 2011; Yang and Leskovec 2013; Luo et al., 2021; Rostami et al. 2023). It is an extension of the widely used Matrix Factorization (MF) method, which decomposes a matrix into two lower-rank matrices (Paatero & Tapper 1994). PMF, however, introduces non-negativity constraints to ensure that the decomposed matrices contain only positive values, making it particularly suitable for analysing non-negative data.

PMF is commonly employed in collaborative filtering-based recommender systems, which aim to predict user preferences and make personalized recommendations (Paatero et al. 2014). In this context, PMF represents users and items as vectors in a low-dimensional latent space, where the dot product between the user and item vectors estimates the preference or rating. By decomposing the user-item rating matrix into user and item latent feature matrices, PMF learns the latent factors that capture the underlying patterns and relationships in the data. The latent factors learned by PMF can often be interpreted as meaningful features, providing insights into the underlying characteristics of users and items.

Compared to standard questionnaire evaluation techniques, the PMF data processing procedure offers several distinct advantages. One notable advantage is its capability to capture individual respondent characteristics rather than solely focusing on mapping relationships between answers. This enables a more comprehensive understanding of each respondent’s unique preferences or attributes.

An important consideration when opting for the PMF methodology is its ability to handle incomplete fillings without complications during the modelling process. This is an essential requirement when assessing questionnaires that have not been fully filled out, a situation that applies to our survey as well.

However, like other matrix factorization methods, PMF can be susceptible to overfitting if the latent dimensionality is chosen to be too high or the regularization is not appropriately tuned. Therefore, during modelling, it is very important to have preliminary expectations to evaluating the results.

In summary, our complementary data evaluation process aimed to go beyond traditional evaluation methods by seeking to identify distinct attitudes and determining their significance among the respondents. In addition to the standard evaluation metrics, we wanted to gain insights into the diverse characteristics and perspectives of individuals, allowing us to understand their varying influence and impact.

Data evaluation using the PMF technique (US EPA version 5.0, ref. PMF 2023) was considered for nearly all questions of the survey (except background questions, Q23-25, and questions related to previous knowledge about PIANOFORTE, Q5-6).

## Results and discussion

### Individual evaluation of questions

#### Basic characteristic of responding population

A total of 440 respondents answered the survey and the answers formed the basis for the individual evaluation of questions. Since answering was not mandatory in all cases, only those respondents who answered the given question are taken into account in the statistical evaluation (otherwise, we emphasize it separately). The gender of the respondents in the survey was not considered. The age of respondents was in the range 18–90 + years with a clear prevalence in the range 40–59 years.

#### Country of residence

The survey gathered the responses of participants from a total of 34 different countries, including 29 European countries, as well as Canada, China, Colombia, India, and the United States. The inclusion of these international responses is particularly useful as it provides valuable global information and enables some comparisons.

**Fig. 1:**
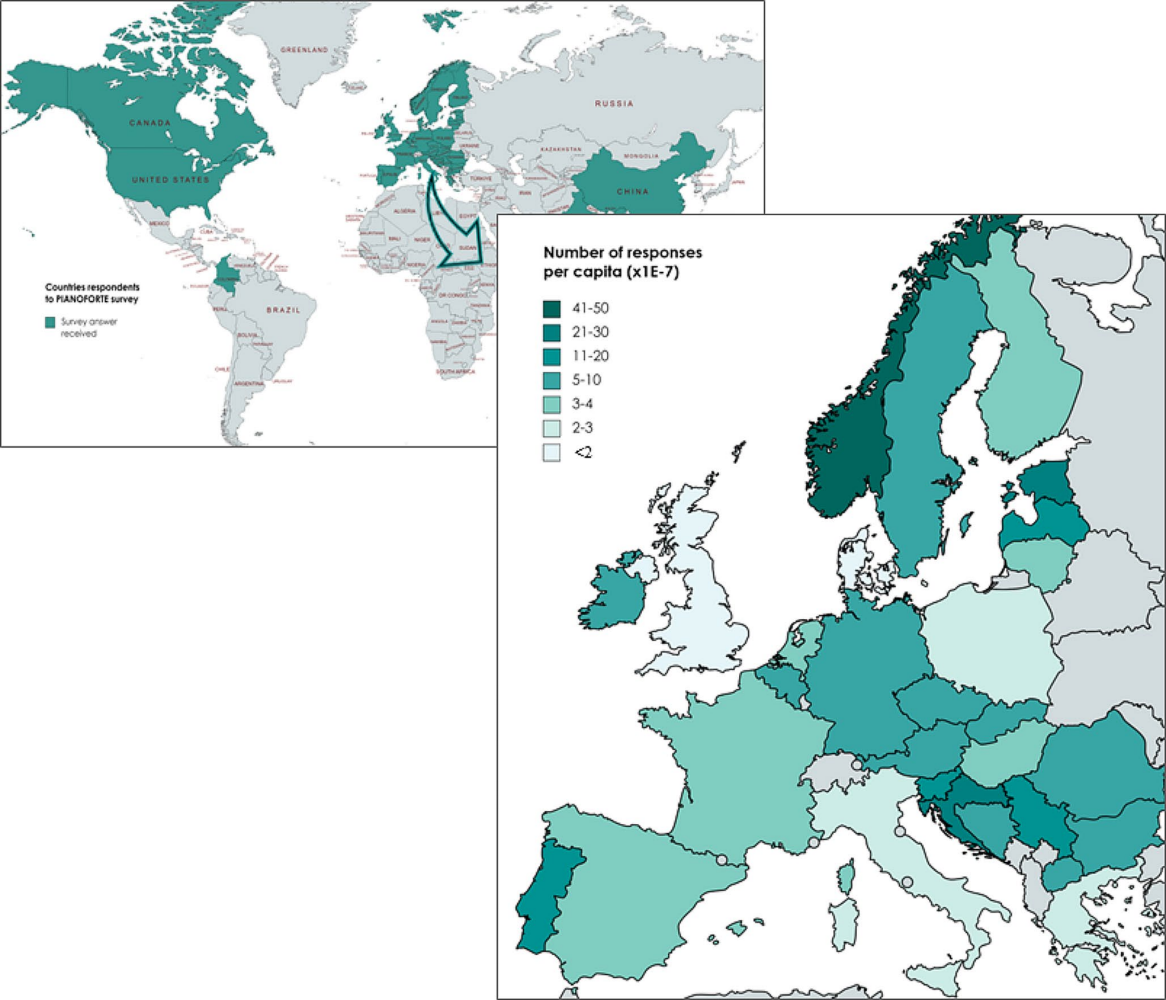
Countries of respondents worldwide and distribution of the respondents across the European countries

Figure 1 provides an overview of all countries that participated in the survey, and the number of responses per capita received from different European countries. Notably, Germany, Norway, France, Spain, and Italy recorded the highest number of responses. However, when considering the number of replies per million citizens, the response rates across countries were relatively homogeneous. Some countries, such as Norway, Croatia and Estonia exhibited somewhat higher response rates considering their respective population sizes. It is worth mentioning that approximately one-third of the respondents, unfortunately, did not provide information regarding their country of residence, impacting the distribution presented in Fig. 1.

#### Respondent stakeholder groups

The analysis of the respondent’s profession showed a wide range (see Table 1.), including researchers, regulators, experts from international organizations and associations (IAEA, ICRP, UNSCEAR, IRPA, ENA, ERA, etc.), engineers, inspectors, legal advisors, medical doctors (radiologists, radiation epidemiologists), medical physicists and radiographers, managers, social scientists and general public not working with any aspect of radiation protection. The largest group of respondents were researchers and representatives of the Education & Training Community (39.8%), followed by national regulators (27.9%), implementers and users of ionising radiation applications (24.6%). Civil society organisations are somewhat under-represented among participants (8.2%).

**Table 1:** Response rates to the following question: please mark below which of the identified stakeholder groups you belong to, or you can identify with

Answer Choices	Responses (%)
Stakeholder and Advisory Board of PIANOFORTE (Internal stakeholders of PIANOFORTE)	5.7
International organisations – European policy makers (EC, Article 31 Group of Experts, HERCA, WENRA and others)	2.5
International organisations and associations – Experts in RP and other related disciplines (IAEA, ICRP, UNSCEAR, IRPA, ENA, ERA and others)	11.9
National policy makers and regulatory authorities – ministries, regulatory bodies, including regional and municipal levels - from different EU countries	28.0
Implementers/Users – national representatives from nuclear industries, non-nuclear industries, trade organisations, medical professional associations in hospitals, national associations on RP, waste management organizations, RP experts, RP officers, medical, technical, scientific instruments manufacturers	24.6
Research and Education & Training Community – research centres, universities, institutes, research platforms on other topics than RP/use of ionising radiation	39.8
Civil society and affected communities – national, regional, local public organizations gathering impacted public groups, or other thematic groups including but not limited to medical patients’ organisations, including individual patients, citizens (e.g., citizen science networks, representatives of communities living in areas near legacy sites and of municipalities with nuclear facilities)	4.8
NGOs – focusing on different topics	2.3
Media – journalists, persons working in communication area and other media	1.1
Metrology – manufacturers of ionising radiation measuring devices; national metrological institutes (NMIs), EURAMET, calibration, certification and quality management (ILAC) organisations	5.1
Participant of PIANOFORTE	15.0

#### Stakeholders’ opinions on radiation protection research topics for future Research and Development (R&D) open calls

To obtain an overview of stakeholders’ opinions on radiation protection topics that should be prioritized in future R&D calls, the respondents were asked to rank each topic by assigning a score on a scale from 1 to 8, representing minimal to maximal importance. Below, the topics based on the EJP CONCERT Joint Roadmap, are ranked from highest to lowest score, with the average ranking, the median of answers and the number of respondents, respectively, given in parentheses:

1. *Understanding and quantifying the health effects of radiation exposure* (6.6, 7, 291).
2. *Optimising medical use of radiation* (6.3, 7, 290).
3. *Improving radiation protection of workers and population* (6.1, 6, 293).
4. *Optimising emergency and recovery preparedness and response* (6.0, 6, 286).
5. *Developing an integrated approach to environmental exposure and risk assessment from ionising radiation* (5.9, 6, 293).
6. *Radiation protection in/with society* (5.4, 5, 287).
7. *Improving the concepts of dose quantities* (5.3, 5, 286).
8. *Understanding radiation-related effects on non-human biota and ecosystems* (4.8, 4, 284).

The ranking of research priorities shows that overall, the scientific foundations of protection of human beings are considered by participants to be most important among the respondents to the survey while the importance of protection of overall non-human biota, ecosystems and biodiversity had the somewhat lower average score. This suggests that the anthropocentric view (as opposed to the ecocentric one) is still dominant for many national regulators (HERCA, 2021) and industry operators. This result may further suggest that the ongoing community discussions such as in ICRP (International Commission on Radiological Protection) on this issue and proposed activities further an integrated, holistic approach (e.g. SRA agenda of the Radioecology ALLIANCE, Gilbin et al. 2021) should be continued with involvement of different parties in radiation protections (e.g., experts from international organisations, but also national regulators, managers and industry operators, researchers).

#### Opinions on most important improvements needed concerning radiation protection

Respondents were asked for their opinions on the most important improvements needed in the future, concerning radiation protection. The possibility for selecting multiple answers among a predefined list was given. The topic of ‘*Research and development and their relationship to regulatory and management practice’* was the most selected option, with 63% of respondents assigning this a high importance. It was closely followed by the ‘*International collaboration in the field of radiation protection’* (60%). These two options, likely reflected the large fraction of researchers (40%) among the respondents. The third most selected response was ‘*Management practices in different countries concerning radiation protection’* with 42% of respondents, followed by ‘*Regulatory approaches in different countries concerning radiation protection’* (35%) and ‘*Legislative requirements for radiation protection’* (22%).

#### Areas of radiation protection and application of ionising radiation of greatest concern and/or involvement

To explore the more general opinion of stakeholders on major radiation protection issues, participants were asked to mark the *‘Areas of radiation protection and application of ionising radiation that are of potential concern and/or subject to involvement for them’* (again, multiple choice was allowed). The chosen answers were as follows:

- *Environmental radioactivity and radioecology* (44%),
- *Naturally occurring radioactive materials (NORM) including radon* (42%),
- *Use of ionising radiation in medical diagnostics or treatments* (41%),
- *Emergency preparedness and recovery* (34%),
- *Use of ionising radiation in research* (30%),
- *Radioactive waste or spent nuclear fuel and decommissioning* (25%), and.
- *Use of ionising radiation in nuclear industry*,* nuclear power plants* (20%).

The responses related to environmental and radioecological issues in RP, NORM and radon, and medical application of ionising radiation were those of higher concern or subject of involvement for the stakeholder respondents. It’s noteworthy that in response to inquiries about future research project priorities (Subsection 3.1.4), the subjects of radioecology and environmental radioactivity, including the examination of non-human biota, received lower scores. Ultimately, answers can be construed as shedding light on the areas where the appreciation of their significance is reasonably satisfactory, but also highlighting the imperative for continued efforts to minimize potential doses to both humans and the environment, as well as addressing potential long-term pollution issues.

Furthermore, a significant proportion of respondents in this survey can be classified as RP experts or professionals, which may explain the currently ‘low ranking’ of concern for nuclear power plants, which historically have been subject of public concern (Eurobarometer 2009) but less so for RP experts. Previous research has proven that there are clear differences in risk perception of these two groups (Burns and Slovic 2012; Perko 2014). Moreover, the past few decades have revealed a heterogeneous landscape of changing public attitudes towards nuclear power across European countries. This diversity is underscored in previous reports (Europeans and Nuclear Safety in 2006 and 2009, Eurobarometer 2006 and 2009). Some European nations exhibit a more favourable public stance on nuclear power, with over 50% expressing positivity in countries like Sweden, the Czech Republic, and Slovakia. In contrast, others, such as Greece, Cyprus, and Portugal, register a positive attitude in the 10–20% range. This emphasizes the significant influence of factors like respondent demographics (where public opinion may diverge from expert views), a country’s historical engagement with nuclear power, and global developments on shaping these perspectives.

#### Medical exposure

A dedicated question was asked ‘*Which of the following medical applications of ionising radiation do you consider as the highest concern/risk with respect to received radiation dose’.* This aimed at exploring the concerns regarding medical exposures. A not negligible fraction of survey respondents (288 in this particular question) answered either ‘*I do not consider any medical applications of ionising radiation to be of concern and potential risk’* (8%) or ‘*I don’t know/not applicable’* (18%). For the rest of the respondents, ‘*Use of ionising radiation in therapeutic purposes’* (22%), ‘*Interventional radiology’* (20%), and ‘*Diagnostic CT’* (17%) are dominating reasons of concern, notwithstanding the benefit that is always assumed according to the justification principle of radiation protection. Medical procedures ‘*PET-CT*,* X-ray imaging* and *Scintigraphy* ‘were at the bottom of the list, while no one selected procedure of ‘*Mammography’* to be a reason of potential concern for radiation protection.

#### Nuclear safety and security

The issue of nuclear safety and security was raised by asking respondents’ opinion on nuclear power plants (NPP), but also on nuclear threats and emergencies. The use of NPPs is supported by the majority of respondents (73%) who agreed with the statements that they are *‘Highly valuable sources of energy with low carbon footprint*,* so their work should be supported’*, and that ‘*With the threat of climate change*,* nuclear energy complements renewable energy and still cannot do without it’*. Although with large country heterogeneity among the EU population, as mentioned in Sect. 3.1.6. (Eurobarometer 2006 and 2009), there is a notable increase towards favouring nuclear power, as evidenced by a substantial 40% approval rate in 2016 (Századvég 2016). However, this study considering a sample largely consisting of RP professionals, showed a significantly higher percentage of support than observed when considering a representative sample of general public in EU countries.

Regarding emergency situations of highest concern, ‘*Incidents and accidents (including criticality accidents) in nuclear installations (power generation*,* research reactors*,* etc.)’* was highlighted by more than a third of respondents (35%). Besides that, *‘Terroristic threats involving radioactive material/ionising radiation’* was selected by 22% of respondents, followed by ‘*Military installations and operations (including submarines)’* (13%). Additionally, regarding security concerns, it is important to note that the ‘*Potential scenario of using nuclear weapons in war in Ukraine’* was listed as a significant concern in the field ‘*Other’*.

The analysis of responses related to stakeholders’ views on issues related to radioactive waste and decommissioning showed that the dominant concern in these cases was the potential ‘*Radioactive pollution and related health and environmental issues for future generations’*, chosen by almost every second survey participant (45%). More than 30% selected the options ‘*Exposure due to a radioactive release in disposal sites and/or decommissioned facilities’* and ‘*Future land use at places that were disposal sites or at decommissioned facilities’* as the most important issues.

#### Naturally occurring radionuclides as potential radiation risk sources

The majority of survey respondents were aware that naturally occurring radionuclides are a source of potential radiation risk under certain industrial or environmental conditions (91%). A number of worldwide publications investigating different aspects of naturally occurring radionuclides (NOR) and conditions for their mobilisation or accumulation, effects and dose related impacts are available nowadays (Cagno et al. 2020; Mrdakovic Popic et al. 2014; Rosen et al. 2012; Turtiainen et al. 2013). Particularly high awareness was expressed about radon gas (98%), which could be expected given the particularities of the respondents. Different sources of information on radon are listed in the responses, such as media, social media, authorities, but academia is listed as the most important one. The EU BSS requirement for implementation of the National Radon Action Plan and intensive activities related to radon level reduction and risk communication in different countries (Bochicchio et al. 2022) probably also contributed to the high expressed awareness. However, less than third of all respondents (30%) acknowledged having performed a radon measurement at home.

#### Matrix questions

**Fig. 2:**
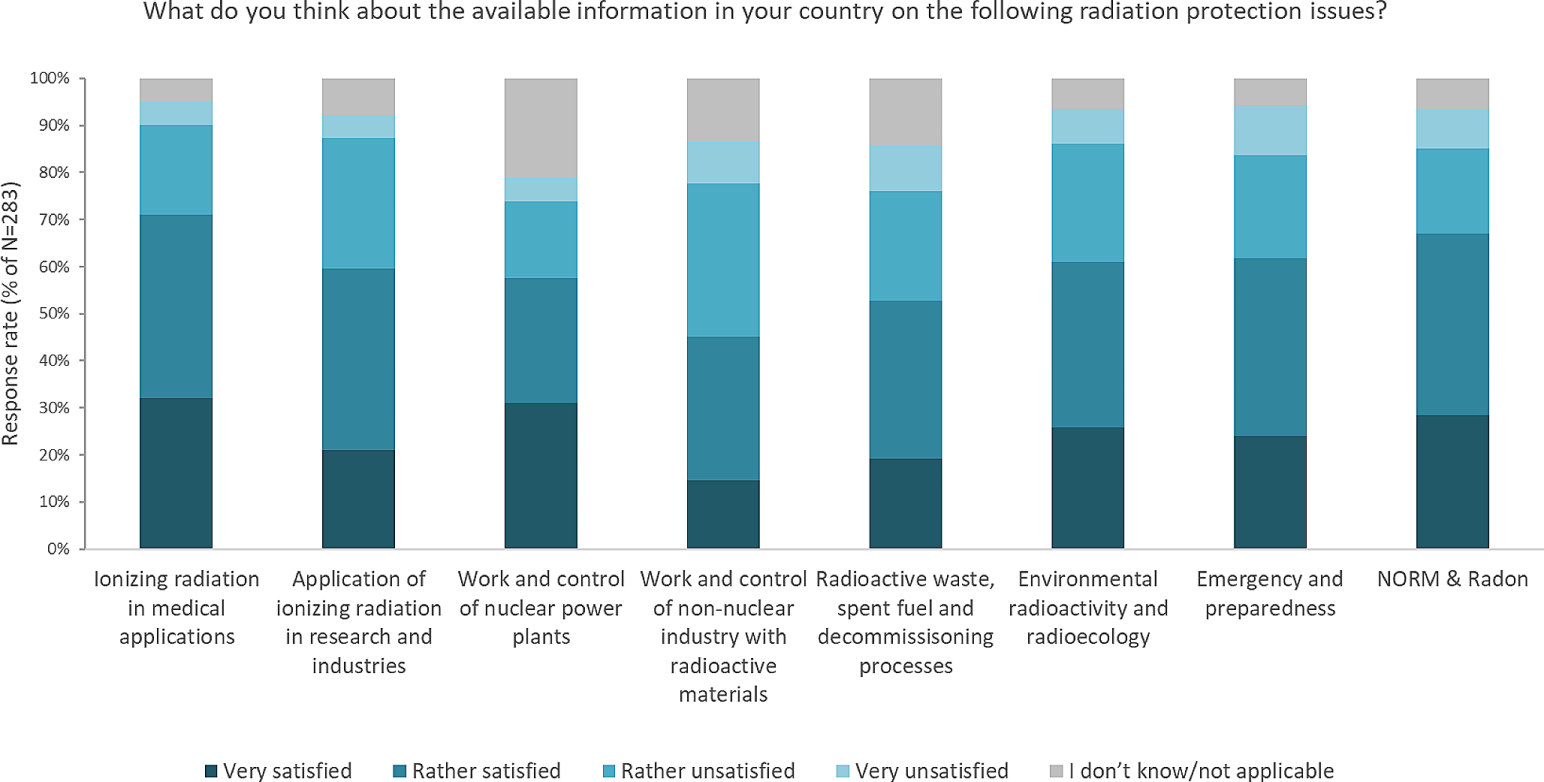
Statistics of respondents’ satisfaction ratings with the available national information on different RP issues

**Fig. 3:**
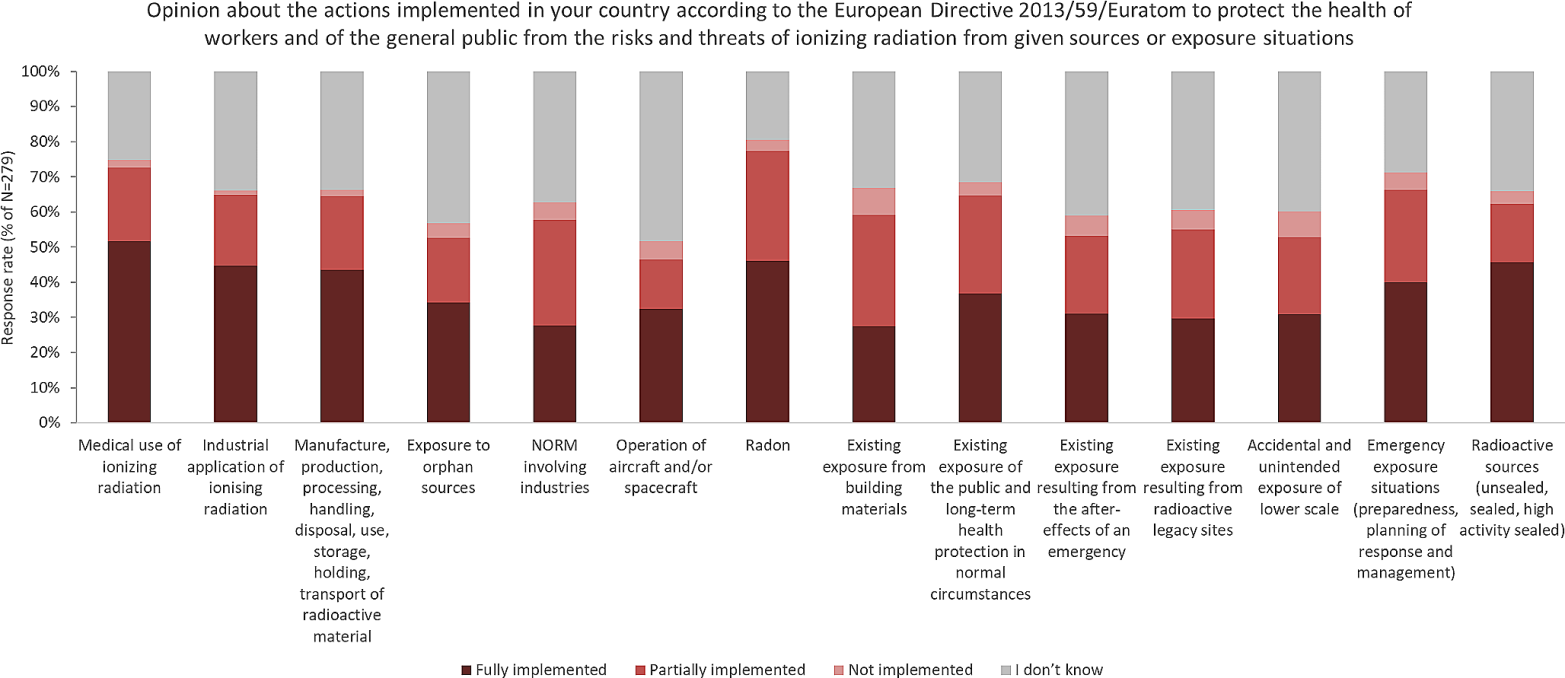
Statistics of respondents’ opinion about the level of implementation of measures required by the EU Basic Safety Standard Directive 2013/59 (EU BSS) for specific RP issues

Finally, two so-called matrix questions were asked, where respondents could express their (a) satisfaction with the available national information on different radiation protection issues (see detailed results on Fig. 2), and (b) opinion about the level of implementation of measures required by the EU Basic Safety Standard Directive 2013/59 (EU BSS) for specific radiation protection issues [EC, 2014] (see Fig. 3).

Based on responses for these two matrix questions, it could be concluded that:

- The level of satisfaction with publicly available information on the given topics of radiation protection is the highest for ‘*Ionising radiation in medical applications’* (71% of respondents answered that they are either rather or very satisfied). A high level of satisfaction (61–67% of respondents) was also expressed for available information on *‘NORM and radon’*, ‘*Emergency and preparedness’* as well as for ‘*Radioecology and environmental radioactivity’*.
- In contrast, the lowest levels of satisfaction were expressed with regard to the information available on ‘*Work and control of non-nuclear industry with radioactive materials’* and regarding *‘Radioactive waste*,* spent fuel and decommissioning’ (41 and 33% respectively)*.
- About 63% of all respondents answered the questions on the level of implementation of EU BSS requirements related to the specific topics of radiation protection. The range of those who answered ‘*I don’t know*’ was 19–44% depending on the question about given radiation sources or exposure situations, most likely reflecting quite a few different stakeholder groups respondents on the survey that might not be in a position to answer this rather regulatory questions.
- The highest percentage of respondents expressed the opinion that the requirements from EU BSS are fully implemented in case of ‘*Medical use of ionising radiation’* (51.6%), *‘Radon’* (46.2%), *‘Radioactive sources and Industrial application of ionising radiation’* (45.7%).
- Requirements of the EU BSS related to the *‘Existing exposure from building materials’*,* ‘Exposure to Orphan sources’ or ‘Accidental and unintended exposure of lower scale’* are those that require better implementation (or marked as not implemented) according to the respondents of this survey.

The statistical analysis of responses with respect to different countries was difficult due to the limited number of responses for certain countries and for some of the given EU BSS requirements. However, an illustrative example of most frequent responses from all stakeholders’ groups/country are presented in Table 2, regarding implementation of the EU BSS requirements related to ‘*Medical use of ionising radiation’* and ‘*Exposure to orphan sources’*. These two types of requirements in the analysis of pooled responses had, as mentioned above, the highest and lowest percentage of implementation, respectively (Fig. 3). From the available stakeholder responses in Table 2 it can be observed that in most European countries, implementation activities regarding ‘*Medical use of ionising radiation’* and ‘*Exposure to orphan sources’* are either fully or partially implemented. However, there were also a number of countries expressing ‘*I don’t know*’ opinion regarding implementation of requirements regarding ‘*Exposure to orphan sources’*, while this was not the case for *‘Medical use of ionising radiation’*. It must be highlighted that these results should be understood as a snapshot of the opinions of this particular survey respondents, belonging to different stakeholder groups, and not an official analysis of the implementation levels of EU BSS requirements in the given countries. However, they could indicate the better level of general information and understanding of nationally developed regulatory approaches and control for ‘*Medical use of radiation’* than for ‘*Exposure to orphan sources’.* A statistical analysis that would ensure the exact view into the level of implementation of all particular EU BSS requirements/countries is planned in future information collection from more specific target groups (e.g. national regulators).

**Table 2:** The most frequent country specific opinion of respondents on questions about the implementation level of EU BSS requirements in their national legislations, EU MS and non-EU MS. An illustrative example of response heterogeneity

Opinion	Requirements regarding Medical use of ionising radiation	Requirement regarding Exposure to Orphan Sources
Fully implemented	Austria, Belgium, Bulgaria, Croatia, Czech Republic, Estonia, Finland, France, Germany, Greece, Ireland, Italy, Latvia, Lithuania, Norway, Portugal, Romania, Slovakia, Slovenia, Spain, Sweden, United Kingdom of Great Britain and Northern Ireland	Belgium, Bulgaria, Croatia, Czech Republic, Estonia, Finland, Greece, Hungary, Ireland, Italy, Latvia, Lithuania, Portugal, Romania, Slovakia, Slovenia, Spain, United Kingdom of Great Britain and Northern Ireland
Partially implemented	Bosnia and Herzegovina, Hungary, Poland, The former Yugoslav Republic of Macedonia	Bosnia and Herzegovina, Serbia
Not implemented	Serbia	-
Don’t know	Denmark	Austria, Denmark, France, Germany, Norway, Poland, Sweden, The former Yugoslav Republic of Macedonia

### Group evaluation of questions

#### Grouping of stakeholders by their opinion

Given the design choice that we did not know beforehand what type of respondents would participate in the survey, we aimed to identify groups with similar interests in the analysis phase. We employed the PMF methodology to identify distinct types or attitudes of stakeholders within our analysis. Throughout the modelling process, the modelled factors were defined as individual stakeholder groups, whose item number (factor) has to be predefined. We conducted modelling experiments considering different numbers of factors, ranging from three to seven, of which the most interpretable was found for a 4-factor solution.

In the following, we provide a brief summary of the characteristics obtained based on the answers of the four resulting groups. Those respondents who answered very few questions - typically only the first couple of questions - were not taken into account during the evaluation, which means that 299 of the total 440 respondents are included in this analysis.

**Group1 (n = 48)** The members of this group mostly belong to the Research and the Education & Training Community. This group focuses primarily on researching the impact of radiation on non-human biota and ecosystems, and with a particular interest in radioactive waste and decommissioning. They do not express concerns about medical uses of ionising radiation or nuclear power plants. Their main worries revolve around radioactive waste repositories, transport accidents involving radioactive materials, and future land use at disposal sites or decommissioned facilities. They show dissatisfaction with the information availability in their country related to radiation protection issues and their own country’s implementation efforts. This group responded selectively or chose more often the ‘I don’t know/ not applicable’ category than other groups.

**Group2 (n = 84)** The group includes professionals representing policy makers, regulatory authorities, NGOs, media, metrology organizations and is the only group that includes members of civil society. They emphasize stakeholder involvement in PIANOFORTE and focus on comprehensive reporting of research results, facilitating discussions, and staying informed about project outcomes. Their priority concerning R&D projects covers health effects of radiation, optimizing medical radiation use, integrated environmental exposure assessment, emergency preparedness, and improved radiation protection. They emphasize legislative requirements, international collaboration, and links between research and development and regulation as vital radiation protection improvements. They worry about medical use of ionising radiation, highlighting concerns in diagnostic CT, PET-CT, and scintigraphy. Only 16% find nuclear power plants acceptable, while 28% advocate reduction and 36% view them as risky. This suggests that the group with civil society ties is more cautious than the other expert groups and might be closer – for these items - to the general acceptance rate in the wider public. Military installations and incidents in nuclear installations are seen as top emergency risks. Respondents from this stakeholder group are concerned about waste and decommissioning, focus on radioactive pollution, and future health and environmental effects. They acknowledge natural radionuclides and radon, but do not often measure them at home. Dissatisfaction is expressed with country-specific radiation protection and local implementation efforts.

**Group3 (n = 108)** This group represents a diverse group of stakeholders, including international organizations, policy makers, ministries, researchers, education communities, NGOs, and metrology experts. Their involvement in PIANOFORTE is to influence research priorities for radiation protection, seeking active participation in projects of mutual concern. They prefer consultation and participation over mere information dissemination. They would like to provide insights on Open Calls, long-term objectives, and research priorities. Concerning ionising radiation, they worry about various applications except medical use, which they find unproblematic. For the reason of climate change concerns, they accept nuclear power plants, and identify risks such as lost sources, military operations, satellite re-entries, and uncontrolled spread of radioactivity. Worries related to waste and decommissioning focus on potential exposures from disposal sites or decommissioned facilities. They acknowledge the presence of natural radionuclides but they believe that it is not always controllable. It is common among them to measure radon at home. They express dissatisfaction with country-specific radiation protection and local implementation.

**Group4 (n = 59)** Members of this group represent PIANOFORTE or diverse stakeholders, including international organizations, policy makers, implementers and media professionals. They prioritize understanding health effects of radiation, optimizing medical use, and protection of workers/the population. They emphasize international collaboration and the link between research and regulation as key for radiation protection improvements. Concerns span the use of ionising radiation in medical applications, research, and nuclear industry, with exceptions for X-ray imaging and scintigraphy. Views on nuclear power plants vary, and alternatives are desired. They highlight transport accidents and terror threats involving radiation as top emergencies. Concerns about waste and decommissioning involve proximity to such facilities. They recognize natural radionuclides, and often measure radon at home. They express high satisfaction with country-specific radiation protection and local actions.

Table 3 summarizes the detailed resulting properties of the four stakeholder groups defined based on PMF modelling results.

**Table 3:** Properties of stakeholder groups from various RP aspects

	Group number	Group1	Group2	Group3	Group4
	Number of respondents belonging to group	48	84	108	59
*3.1.3*	***Member of stakeholder group***	Research and Education & Training Community	• National policy makers and regulatory authorities • NGOs • Media • Metrology • Civil society and affected communities	• International organizations • National policy makers and regulatory authorities • Ministries • Research and Education & Training Community • NGOs • Metrology experts	• International organizations • European policy makers • Implementers/users • Media • PIANOFORTE member
3.1.4	***R&D project***	Effects of radiation on non-human biota and ecosystems	• Understanding and quantifying the health effects of radiation exposure • Optimizing medical use of radiation • Develop risk assessment • Optimizing emergency and recovery preparedness and response • Enhancing RP for workers and the general population • RP within society	No significant	• Understanding and quantifying the health effects of radiation exposure • Improving concepts of dose quantities • Optimizing medical use of radiation • Enhancing radiation protection for workers and the general population
3.1.5.	***Improvements in RP***	Not outlined	• International collaboration, • Strengthening the relationship between research and development and regulatory and management practices • Legislative requirements for radiation protection	No significant	• International collaboration • Strengthening the relationship between research and development and regulatory and management practices
3.1.6	***Main concern in RP and application***	Radioactive waste or spent nuclear fuel and decommissioning	• Emergency preparedness and recovery	All except medical	• Medical diagnostics or treatments • Research purposes • Nuclear industry
3.1.7	***Medical applications concern***	None/ I don’t know	• Diagnostic CT • PET-CT • Scintigraphy	None/ I don’t know	• Diagnostic CT • PET-CT • Interventional radiology • Use of ionising radiation in therapeutic purposes
3.1.8	***Attitudes towards NPP***	no opinion	negative	favourable	diverse
3.1.8	***Threats leading to emergency situations***	• Radioactive waste repositories • Transport accidents involving radioactive material	• Incidents and accidents (including criticality accidents) in nuclear installations • Military installations and operations	Several	• Transport accidents involving radioactive material • Terroristic threats involving radioactive material/ionising radiation
3.1.8	***Concern on waste and decommissioning***	Future land use at locations that were used as disposal sites or decommissioned facilities	Radioactive pollution and related health and environmental issues for future generations	Exposure due to radioactive releases in disposal sites and/or decommissioned facilities	• Living in the vicinity of radioactive waste and/or decommissioning facilities • Radioactive pollution and related health and environmental issues for future generations
3.1.9	***Naturally occurring radionuclides or radon***	No opinion	Aware Not often measuring	Acknowledge it but believe that they are not always controllable often measuring at home	Aware Often measuring
3.1.10	***Country-specific RP issues – available information and implementation of actions within their own country***	Low rating	Low rating	Low rating	High rating

#### Evaluation of the applied PMF methodology

To evaluate the grouping, it is necessary to discuss the reliability of the solution (Paatero et al. 2014; Brown et al. 2015). Bootstrap (BS) intervals encompass the effects resulting from random errors, and partially incorporate the effects of rotational ambiguity. In addition, the Displacement intervals (DISP) indicator specifically accounts for the effects of rotational ambiguity. Notably, the DISP swap counts serve as a pivotal metric for gauging the stability of our PMF solution, which in our analysis counts consistently registered at 0, indicating that our results are both interpretable and robust.

Furthermore, the error estimation conducted through the bootstrap method revealed that, among the convergent runs, three factors exhibited mappings exceeding 93%. Conversely, for Group 1, the BS mapping yielded a lower rate of 63%. This discrepancy suggests a potential linkage to other factors, implying that this group may share characteristics with the others. Despite this, we deem it worthwhile to retain Group 1 in our analysis, given the interpretability and value of the results obtained.

Indeed, it is crucial to bear in mind that the stakeholders completing the questionnaire cannot be neatly categorized into the specific groups derived from individual PMF modelling. Instead, they are assigned a “most typical” grouping based on their responses. Consequently, it is valuable to assess to what extent the overall respondent population aligns with each group. Our findings reveal that the properties of the third group are prevalent among the largest (42%) proportion within the respondents. This group exhibits characteristics such as a strong commitment to radiation protection and a desire to act. In contrast, traits associated with groups 1 and 2 are only found in approximately with weight of 16–17% of the respondents.

#### Discussion of group opinions

During the distribution of our questionnaire, our primary objective was to engage a diverse spectrum of stakeholders. We received feedback from nearly every European country, along with participation from five other nations, which means that a broad international response sample has been collected. Keeping in mind that the sample of participants had several categories (e.g. civil society, social science researchers) under-represented, it did manage to capture a large amount of input from stakeholders (70%) who had not previously participated in such surveys.

However, we encountered some challenges during the survey process. Notably, a portion of respondents, approximately a quarter, chose not to answer all questions. This trend was largely attributed to a pattern where many participants discontinued their response after the initial questions. On a positive note, however, at least half of the respondents did provide their names, indicating a high level of engagement and cooperation.

Furthermore, a substantial portion of the respondents demonstrated proactive and cooperative behaviour, only 2% of respondents considered PIANOFORTE ‘Not at all important’. Our analysis of individual responses revealed a variety of topics of interest, reflecting the diversity of stakeholder groups that participated. On the other hand, we could successfully apply the PMF method to group respondents and attitudes. As a result, we identified four distinct opinion clusters. Interestingly, even though only a small number of respondents identified themselves as non-professionals in the field of radiation protection, they predominantly clustered within just two groups (1&2). Given that improving radiation protection is relevant to the broader society, it is crucial to explore the areas of interest and concerns specific to these groups. Members of group 1 frequently responded with ‘I don’t know’ to several questions, indicating a lack of decisive opinions. On the other hand, those in group 2, which as the only one includes members of the broader public (however only 17 individuals) among other stakeholders, exhibited a higher degree of concern. This group displayed the strongest disapproval of nuclear facilities, and notably, radon measurement was infrequently observed among its members. Members of these opinion cluster expressed low satisfaction with the availability of information and the implementation of measures in accordance with the EU BSS. Notably, a significant number of respondents either chose not to answer or lacked a clear opinion on these matters, indicating a potential information gap that needs to be addressed.

Regarding groups 3 and 4, we were able to identify stakeholder groups with a notably positive attitude towards nuclear facilities. A substantial portion (65%) of these individuals have actively conducted radon measurements in their homes, reflecting a higher level of awareness and engagement in this regard.

The formation of opinion groups highlights the diversity of respondents and the need to ensure participation of a wide variety of stakeholders in decisions on radiation protection research, policies, and practices. It is essential to acknowledge that these opinion groups do not necessarily represent the entire international stakeholder community comprehensively. However, they do provide valuable insights and guidance in shaping the direction of our communication strategies and information dissemination channels.

## Conclusions

Overall, the survey provides insights on the current viewpoints of a diverse set of radiation protection stakeholders, mostly from the professional field. However, it should be highlighted that a good number of non-professional stakeholders as well as many newcomers were reached by the survey. The survey cannot be considered as representative in view of either only a general public or expert opinion on radiation protection issues, but the results contribute to a better understanding of the perceived importance of stakeholder involvement and proved the increased need of targeted communication activities. Communication, raising awareness, as well as ensuring opportunities for active participation in shaping radiation protection research and policies, and capacity building through provision of high-quality training and informative material are key and will continue to be in the focus of the radiation protection community within the PIANOFORTE project and beyond.

The survey showed that PIANOFORTE is well known also by many stakeholders from outside the Partnership, suggesting that the Partnerships’ dissemination and communication activities from the first project months have been fruitful. By including informative contents about the Partnership and its activities, the survey has increased awareness of PIANOFORTE and radiation protection related issues and leveraged the number of officially registered stakeholders. The survey respondents’ feedback on which research topics have already been used within the prioritization process of PIANOFORTE for the topics of the first Open Call. Responses were diverse, as anticipated because of the diverse structure and varying professional background of the survey participants. As a whole, the research topics in the field of health and medical use of ionising radiation were considered as having a slightly higher priority. Given the fact that a cross-topic ranking of such diverse fields as emergency preparedness and medicine is by construction subjective, this survey’s feedback gives an external reality check to the discussion within the organized scientific community and PIANOFORTE.

Going from research to implementation, an important finding from the survey is the substantial heterogeneity in the perception of EU BSS implementation. This highlights the imperative for comprehensive follow-up analysis in this domain. We raise the question of whether involving only regulatory experts is sufficient for the successful implementation of these standards, emphasizing the importance of ongoing monitoring efforts.

The most important limitation of the current study is that the overall number of respondents is limited in size and they have been chosen randomly but biased through distribution channels, precluding it from being deemed representative, however it is worth noting that participants hail from numerous countries. As a result of this and numerous data gaps, the scope for a comprehensive statistical assessment is constrained. A broader pilot study, including more representatives of the target population from different countries and cognitive interviews, could have identified issues earlier. However, despite these limitations, the survey was found to be a valid method for obtaining an initial overview of the research area and generalizing this information.

Based on the current survey outcomes, the next survey is planned for 4–5 years from now, towards the end of the PIANOFORTE partnership, similar to the timing of this survey conducted four years after the previous one. We anticipate continuing intensive communication with stakeholders established so far and further strengthening these interactions. The authors also foresee that new knowledge and outcomes from PIANOFORTE activities, as well as new projects like PREDICT, DISCOVER, and SONORA approved through Open Calls, will provide updated information to professionals in radiation protection and the general public. Therefore, the next survey could focus on assessing whether there is a better understanding of radiation protection issues and identifying new topics relevant to broader groups for future projects. Consideration will be given to structuring future surveys with specific sections for regulators, researchers, and the general public to ensure that questions are tailored appropriately and minimize overlap, such as queries primarily addressed by regulators (e.g., opinions on implementing EU BSS requirements in national legislative frameworks).

To improve the usability of this approach, we recommend continuous refinement of this questionnaire and the establishment of a periodic cycle for reanalysis, ideally every five years. This would serve as a valuable, albeit non-representative, source of informative insights into the implementation of radiation protection measures in Europe – and shifting foci for research that benefits society. It will be a question of funding and organisation to see if a future, regular series of surveys like this one can be organised in a representative way.

### Electronic supplementary material

Below is the link to the electronic supplementary material.

Supplementary Material 1

## Data Availability

The data related to the present study can be obtained upon personal request.
