# Supplementary material for: Quantitative stakeholder-driven assessment of radiation protection issues via a PIANOFORTE online survey
Source: Radiat Environ Biophys. 2024 Jul 17;63(3):307–22. doi: 10.1007/s00411-024-01084-1 (PMC11341616; doi:10.1007/s00411-024-01084-1)
Supplement: Supplementary file 1 — Supplementary Material 1 [file 411_2024_1084_MOESM1_ESM.docx]

**Supplementary Material**

**Quantitative stakeholder-driven assessment of radiation protection issues via a PIANOFORTE online survey**

Veronika Groma^1,*^, Balázs Madas^1^, Florian Rauser^2^, Mandy Birschwilks^2^, Andreas Blume^2^, Almudena Real^3^, Rein Murakas^4,5,6^, Boguslaw Michalik^7^, Isabel Paiva^8^, Tone-Mette Sjømoen^9^, Alan H. Tkaczyk^10^, Jelena Mrdakovic Popic^9^

^1^Environmental Physics Department, HUN-REN Centre for Energy Research, Budapest, Hungary

^2^Federal Office for Radiation Protection, BfS, Germany

^3^Research Centre on Energy, Environment and Technology, CIEMAT, Madrid, Spain

^4^Faculty of Social Sciences, University of Tartu, Tartu, Estonia

^5^Faculty of Arts and Humanities, University of Tartu, Tartu, Estonia

^6^Rein Murakas Consulting, Tartu, Estonia

^7^Silesian Centre for Environmental Radioactivty, Central Mining Institute, Katowice, Poland

^8^Center for Nuclear Sciences and Technologies, Department of Nuclear Engineering and Sciences, Instituto Superior Técnico, University of Lisbon, Portugal

^9^[Norwegian Radiation and Nuclear Safety Authority](https://www.researchgate.net/institution/Norwegian_Radiation_and_Nuclear_Safety_Authority?_tp=eyJjb250ZXh0Ijp7ImZpcnN0UGFnZSI6InByb2ZpbGUiLCJwYWdlIjoicHJvZmlsZSJ9fQ), Oslo, Norway

^10^ University of Tartu, Institute of Technology, Ravila 14a EE-50411, Tartu, Estonia

^*^correspondence: olahne.groma.veronika@ek.hun-ren.hu

**QUESTIONS OF THE SURVEY**

|  |
| --- |

**Introduction**

This electronic survey (e-survey) is organized by researchers from the PIANOFORTE partnership (‘Partnership for European Research in Radiation Protection and Detection of Ionising Radiation: Towards safer use and better protection of the environment and human health’ (2022-2027)) as a part of stakeholder engagement activities.

 The main survey objectives are:

To present PIANOFORTE, its aims and activities to a wide range of stakeholders in European countries,

To explore opinions and views on main radiation protection issues of relevance to stakeholders.

You are kindly invited to voluntarily participate in this e-survey. Estimated time for completing the survey is 15 minutes.

 All collected information will be used exclusively for the scientific PIANOFORTE project purposes, analysed anonymously as aggregated data, and confidentially stored in accordance with the General Data Protection Regulation (GDPR). No personal information will be shared and data will be deleted after the analysis has been completed. The results and main survey conclusions will be presented in a report that will be available to all PIANOFORTE stakeholders.  More information on the PIANOFORTE partnership and Stakeholder engagement (Work Package 3) is available on the *[PIANOFORTE website](https://pianoforte-partnership.eu/stakeholders)*.

#### *** 1. Please mark that you have read and understood the above information and participation conditions.**

Yes

|  |
| --- |

**General questions**

**2. Please mark below which of the identified stakeholder groups you belong to or you can identify with:**

Stakeholder and Advisory Board of PIANOFORTE

International organisations – European policy makers (EC, Article 31 Group of Experts, HERCA, WENRA and others)

International organisations and associations – Experts in radiation protection and other related disciplines (IAEA, ICRP, UNSCEAR, IRPA, ENA, ERA and others)

National policy makers and regulatory authorities – ministries, regulatory bodies, including regional and municipal levels - from different EU countries

Implementers/Users – national representatives from nuclear industries, non-nuclear industries, trade organisations, medical professional associations in hospitals, national associations on radiation protection, waste management organizations, radiation protection experts, radiation protection officers, medical, technical, scientific instruments manufacturers

Research and Education & Training Community – research centres, universities, institutes, research platforms on other topics than radiation protection/use of ionising radiation

Civil society and affected communities – national, regional, local public organizations gathering impacted public groups, or other thematic groups including but not limited to medical patients’ organisations, including individual patients, citizens (e.g., citizens science networks, representatives of communities living in areas near legacy sites and of municipalities with nuclear facilities)

NGOs – focusing on different topics

Media – journalists, persons working in communication area and other media

Metrology – manufacturers of ionising radiation measuring devices; national metrological institutes (NMIs), EURAMET, calibration, certification and quality management (ILAC) organisations

Participant of PIANOFORTE

**3. Have you previously participated in radiation protection surveys as part of projects or other activities?**

No

Yes

If yes, in which?

**4. What is your involvement in radiation protection?**

Professional

Non professional

|  |
| --- |

**Questions related to PIANOFORTE**

**5. Have you previously heard of the European PIANOFORTE partnership?**

No

Yes

**6. If yes, from which source?**

Website

Twitter

Facebook

E-mail

I am a PIANOFORTE partner

Other (please specify)

**7. How important is the stakeholder involvement in the PIANOFORTE project to you?**

Very important

Important

Somewhat important

Not at all important

I don’t know/not applicable

**8. Please mark those activities for which, in your opinion, it is most important to involve different stakeholder groups:**

Participation in local public meetings on specific radiation protection issues together with researchers and authorities to consider situations of concern

Individual interviews with public stakeholders on identified situations of radiation protection concern

To give opinion on radiation protection areas (e.g. medical use of ionizing radiation, emergency and preparedness, radon, etc.) including research topics that should be prioritized in the EU scientific projects

To give opinion on research priorities in strategic research agendas for radiation protection for the next 10 years

To be included as participant in research projects where the topic is of mutual concern and outcomes can be improved through such engagement

To get reported results/outcomes from research projects in a more comprehensive way – and discuss the results achieved in the project

I don’t know/not applicable

Other (please specify)

**9. How would you like to be involved in the stakeholder activities of the PIANOFORTE project?**

By answering this survey

To be consulted to give your opinion on the research priorities identified for the Open Calls that will be organised within PIANOFORTE

To be consulted to give your opinion on long-term research objectives and priorities that will be identified in Strategic Research Agendas and Joint Road Maps of the radiation protection platforms

To be informed about PIANOFORTE results

To actively participate in the discussion and dissemination of PIANOFORTE activities and results

I don’t know/not applicable

Other (please specify)

|  |
| --- |

***10. In your opinion, which topics should be prioritised in joint research and development projects in future? (mark the research priorities from 1-8, lowest to highest priority)***

| Understanding and quantifying the health effects of radiation exposure |  |
| --- | --- |
| Improving the concepts of dose quantities |  |
| Understanding radiation-related effects on non-human biota and ecosystems |  |
| Optimising medical use of radiation |  |
| Improving radiation protection of workers and population |  |
| Developing an integrated approach to environmental exposure and risk assessment from ionising radiation |  |
| Optimising emergency and recovery preparedness and response |  |
| Radiation protection in/with society |  |

**11. In your opinion, the most important improvements needed in the future in our societies, concerning radiation protection, are related to the following aspects:**

Legislative requirements for radiation protection

Regulatory approaches in different countries concerning radiation protection

Management practices in different countries concerning radiation protection

International collaboration in the field of radiation protection

Research and development and their relationship to regulatory and management practice

Other (please specify)

**General questions on radiation protection**

**12. In which area of radiation protection or application of ionising radiation are you most concerned about or involved in?**

Use of ionising radiation in medical diagnostics or treatments

Use of ionising radiation in research

Use of ionising radiation in non-nuclear industry

Use of ionising radiation in nuclear industry, nuclear power plants

Radioactive waste or spent nuclear fuel and decommissioning

Environmental radioactivity and radioecology

Emergency preparedness and recovery

Naturally occurring radioactive materials (NORM) including radon

I don’t know/not applicable

Other (please specify)

**13. Which of the following medical applications of ionising radiation do you consider as the highest concern/risk with respect to received radiation dose:**

Diagnostic CT (computational tomography scan)

PET-CT

Mammography

X-ray imaging

Scintigraphy

Interventional radiology

Use of ionising radiation in therapeutic purposes

I do not consider any medical applications of ionising radiation as concern/risk

I don’t know/not applicable

Other (please specify)

**14. How do you consider the use of nuclear power plants (NPPs) for energy production?**

NPPs are highly valuable sources of energy with low carbon footprint, so their work should be supported

With the threat of climate change, nuclear energy complements renewable energies and still cannot do without it

The number of NPPs should be reduced by systematic decommissioning in future. Plans should be made at national and international levels

NPPs are dangerous for the present and future generations

I am mostly concerned about NPP accidents regarding their operation and maintenance

I don’t know/not applicable

**15. Which of the following threats that could lead to emergency situations do you consider as of highest concern/risk:**

Incidents and accidents (including criticality accidents) in nuclear installations (power generation, research reactors, etc.)

Radioactive waste repositories

Transport accidents of radioactive material

Lost/orphan sources

Terroristic threats involving radioactive material/ionising radiation

Military installations and operations (including submarines)

Satellite re-entry with radioactive sources

Other events involving the non-controlled exposure or spread of radioactivity (Hospitals, Medical & Industrial Isotope Production Facilities, Space Weather, etc.)

I don’t know/not applicable

Other (please specify)

**16. Which of the following statements related to the issues of radioactive waste and decommissioning is of most concern to you:**

Living in the vicinity of radioactive waste and/or decommissioning facilities

Radioactive pollution and related health and environmental issues for future generations

Future land use at places that were disposal sites or at decommissioned facilities

Exposure due to a radioactive release in disposal sites and/or decommissioned facilities

I don’t know/not applicable

Other (please specify)

**17. Do you consider naturally occurring radionuclides as possible source of radiation risk?**

No, it is natural phenomenon

Yes, but only when enhanced due to human activity

Yes, always, however not always amendable to control.

**18. Have you heard about the naturally occurring radioactive gas radon?**

No

Yes

If yes, indicate the main source of information (media, social media, authorities, academia, other):

**19. Have you ever measured radon at your home?**

No

Yes

**20. What do you think about the available information in your country on the following radiation protection issues?**

Very unsatisfied Rather usatisfied Rather satisfied Very satisfied I don’t know/NA

|  |  |  | |  |  |  |
| --- | --- | --- | --- | --- | --- | --- |
| Ionizing radiation in medical applications |  | |  |  |  |  |
| Application of ionizing radiation in research and industries |  | |  |  |  |  |
| Work and control of nuclear power plants |  | |  |  |  |  |
| Work and control of non-nuclear industry with radioactive materials |  | |  |  |  |  |
| Radioactive waste, spent fuel and decommissisoning processes |  | |  |  |  |  |
| Environmental radioactivity and radioecology |  | |  |  |  |  |
| Emergency and preparedness |  | |  |  |  |  |
| NORM & Radon |  | |  |  |  |  |

**21. Please mark your opinion about the actions implemented in your country according to the European Directive 2013/59/Euratom (EU BSS), to protect the health of workers and of the general public from the risks and threats of ionizing radiation from given sources or exposure situations**

^Not implemented Partially implemented Fully implemented I don’t know^

|  |  |  |  |  |
| --- | --- | --- | --- | --- |
| Medical use of ionizing radiation |  |  |  |  |
| Industrial application of ionising radiation |  |  |  |  |
| Manufacture, production, processing, handling, disposal, use, storage, holding, transport, import to, and export of radioactive material |  |  |  |  |
| Exposure to orphan sources |  |  |  |  |
| NORM involving industries |  |  |  |  |
| Operation of aircraft and/or spacecraft |  |  |  |  |
| Radon |  |  |  |  |
| Existing exposure from building materials |  |  |  |  |
| Existing exposure of the public and long-term health protection in normal circumstances |  |  |  |  |
| Existing exposure resulting from the after-effects of an emergency |  |  |  |  |
| Existing exposure resulting from radioactive legacy sites |  |  |  |  |
| Accidental and unintended exposure of lower scale |  |  |  |  |
| Emergency exposure situations (preparedness, planning of response and management) |  |  |  |  |
| Radioactive sources (unsealed, sealed, high activity sealed) |  |  |  |  |

**22. Do you have or have you had any other types of issues related to radiation protection?**

| **PIANOFORTE - Electronic survey**  **Background questions**  Your e-mail address is requested as a unique identifier to allow the merging of your answers to  this survey and follow-up surveys. Your responses, however, will be analyzed anonymously  as aggregated data. |
| --- |

**23. What is your name?**

**24. What is your profession?**

**25. What is your current age?**

Under 18

18-29

30-39

40-49

50-59

60-69

70-79

80-89

90 or older

***** **26. In what country do you live?**

**27. What is your email address?**
